# Modifying Effects of Resting Heart Rate on the Association of Binge Drinking With All-cause and Cardiovascular Mortality in Older Korean Men: the Kangwha Cohort Study

**DOI:** 10.2188/jea.JE20130101

**Published:** 2014-07-05

**Authors:** Mikyung Ryu, Bayasgalan Gombojav, Chung Mo Nam, Yunhwan Lee, Kimyoung Han

**Affiliations:** 1 Department of Preventive Medicine and Public Health, Ajou University School of Medicine, Suwon, Korea; 2 Institute on Aging, Ajou University Medical Center, Suwon, Korea; 3 Institute for Health Promotion, Graduate School of Public Health, Yonsei University, Seoul, Korea; 4 Department of Preventive Medicine and Public Health, College of Medicine, Yonsei University, Seoul, Korea

**Keywords:** alcohol drinking, binge drinking, cardiovascular disease, mortality, resting heart rate

## Abstract

**Background:**

Although binge drinking and high resting heart rate independently affect cardiovascular and all-cause mortality risk, the combined effect of these two risk factors and their interaction has rarely been studied. This study examined the association between binge drinking and cardiovascular and all-cause mortality and evaluated the potential modifying effect on this association of resting heart rate in Korean men.

**Methods:**

Men aged 55 years or older in 1985 (*n* = 2600) were followed for cardiovascular and all-cause mortality for 20.8 years, until 2005. We estimated hazard ratios (HRs) for cardiovascular and all-cause mortality by binge drinking and resting heart rate using the Cox proportional hazard model.

**Results:**

Heavy binge drinkers (≥12 drinks on one occasion) with elevated resting heart rate (≥80 bpm) had a HR of 2.25 (95% confidence interval [CI], 1.47–3.45) for death from cardiovascular disease and 1.37 (95% CI, 0.87–2.14) for all-cause mortality compared to the reference group (non-drinking and resting heart rate 61–79 bpm). The HRs of dying from cardiovascular disease increased linearly from 1.36 to 1.52, 1.71, and 2.25 among individuals with resting heart rate greater than or equal to 80 bpm within the four alcohol consumption categories (non-drinking, non-binge, moderate binge, and heavy binge), respectively.

**Conclusions:**

Our findings suggest that, among older Korean men, heavy binge drinkers with an elevated resting heart rate are at high risk for cardiovascular and all-cause mortality.

## INTRODUCTION

The overall alcohol consumption in Korea is among the highest in the world, with 14.8 L pure alcohol consumed per capita annually,^1^ and heavy drinking is quite prevalent, with 46.3% of adult males being heavy drinkers.^2^^,^^3^ Studies of drinking patterns have consistently found that binge drinking is a risk factor for cardiovascular and all-cause mortality.^4^^,^^5^ People consuming more than 12 drinks on one occasion had the highest incidence of death from cardiovascular disease (CVD), including hypertension and stroke.^4^^,^^6^ A number of studies have found alcohol consumption to be associated with changes in heart rate and arrhythmias.^7^^,^^8^ Likewise, previous epidemiological studies have shown a strong positive association between elevated resting heart rate and cardiovascular mortality in the general population.^9^^,^^10^ Some studies have reported that electrocardiographic changes may develop after long-term alcohol consumption.^11^^,^^12^

Although binge drinking or high resting heart rate independently affects cardiovascular and all-cause mortality risk, the combined effect of these two risk factors and their interaction has rarely been studied. The effect of high resting heart rate on the association between alcohol consumption and cardiovascular and all-cause mortality remains unclear. We used prospective data from the Kangwha Cohort Study to assess whether or not binge drinking is associated with increased cardiovascular and all-cause mortality over total of 20.8 years of follow-up among Korean men aged 55 years or older with high resting heart rate.

## METHODS

### Data source

Data were from the Kangwha cohort constructed in 1985, the first community-based cohort study in Korea. The overall purpose was to examine the effects of various health-related factors, such as smoking, drinking, anthropometric values, blood pressure measurements, and pulse rate, on the morbidity of various diseases, especially chronic non-infectious diseases, in the elderly. Kangwha County is an island off the west coast of the Republic of Korea, approximately 50 km west of Seoul, with a population of 71 116 in 1993.^6^ In February 1985, 9378 Kangwha County residents were 55 years of age or older. The baseline survey for the Kangwha cohort was conducted in March 1985 by 26 trained interviewers. Of the 9378 potential subjects, 6372 (67.9%; 2724 men, 3648 women) agreed to participate. The study participants were followed until December 31, 2005, with a maximum follow-up of 20.8 years for mortality. Of the 2724 men, those with no information on alcohol consumption (*n* = 15) and heart rate (*n* = 46) as well as those who had a prior history of stroke or coronary heart disease (*n* = 63) were excluded, resulting in 2600 as the final study population. The study protocol was approved by the Institutional Review Board of Human Research of Yonsei University (4-2007-0182).

### Measurements

Alcohol consumption was assessed by asking, “Do you drink alcohol?” Those who answered “yes” to the question were then asked about the frequency of drinking, categorized as daily, almost daily, 2 to 3 times a week, 1 to 4 times a month, or 4 to 12 times a year. The type of alcoholic beverage and the amount of alcohol consumed were assessed by asking “How much (in number of bottles or glasses) of a type of alcoholic beverage do you drink on one occasion?” Up to two types of alcoholic beverages that the participants usually consumed on one occasion were recorded. In 1985, at the time of the survey, *soju* contained 25% and *makkoli* 6% alcohol by volume.^4^^,^^13^ A glass of *soju* was 60 mL, with a bottle containing 360 mL. The standard drink was calculated as the amount of drink for *soju* (volume in mL) × percent by volume of alcohol (%) × density of ethanol. Given that the specific gravity of ethanol is 0.785, the amount of alcohol was 11.8 g in a glass of *soju*, with 70.7 g in a bottle. For *makkoli*, a glass was 300 mL, and a bottle amounted to 1800 mL. Thus, a glass of *makkoli* contained 14.1 g, and a bottle 84.8 g, of ethanol. Other types of beverage were not included in the analysis because of the low frequency of consumption, with only 4 participants reporting drinking beer and none drinking wine.^6^ Among male drinkers 83.9% selected only one type and 16.1% selected two types. Binge drinking was defined as consuming ≥6 drinks of alcoholic beverage on one occasion, with those having ≥12 drinks classified as heavy binge drinkers. Alcoholic beverages consumed most often were *soju*, a distilled beverage native to Korea, similar to *shochu* in Japan, and *makkoli*, an unfiltered fermented beverage made of a mixture of wheat and rice, also native to Korea.

Resting heart rate was measured after at least 10 minutes of being supine by palpating the radial artery over a period of 15 seconds and multiplying by 4. If the rate was unusually fast or slow to count, the test was extended to 60 seconds, with the aid of a stethoscope placed over the heart if necessary. Participants were categorized into three resting heart rate groups, based on the cutoff point defined in previous studies^10^^,^^14^: heart rate ≤60 beats per minute (bpm), heart rate of 61–79 bpm, and heart rate ≥80 bpm.

Deaths occurring between March 15, 1985 and December 31, 1991 were identified from records of burial and death certificates from the administrative branch offices of the local government and through telephone calls or visits to living family members by trained surveyors twice a year. Deaths from January 1, 1992 to December 31, 2005 were matched with death records from the National Statistical Office. For deaths before 1992, personal identifiers were not available for matching with the national death registry. The main outcome variables for this study were death from all-causes and death from CVD, according to the 10th revision of the International Classification of Diseases.

Covariates included age (in years), education (no education/elementary/higher), occupation (agriculture/other), smoking (never, former, 1–19 tobacco/day, and ≥20 tobacco/day), and systolic and diastolic blood pressure (as continuous variables; 27 participants were taking antihypertensive drugs). Body mass index (BMI) was defined as measured weight (kg) divided by height squared (m^2^). In assessing chronic conditions, the participants were asked a question, “Do you have any chronic disease, past accident, or injury due to which you feel uncomfortable in your daily life, including work?” Those who answered with an affirmative to this question were then asked to identify the type of chronic diseases and/or accidents or injuries experienced. In the analysis, chronic disease was dichotomized as “ever” or “never”.

### Statistical analysis

One-way analysis of variance and χ^2^ tests were used to analyze sample characteristics by resting heart rate. Continuous variables are shown as means (standard deviation) and were compared using one-way analysis of variance. Categorical variables are shown as numbers and percentages and were compared using χ^2^ tests for association. The Cox proportional hazard model was used to test the relationship between alcohol consumption and resting heart rate status at baseline and subsequent risk for cardiovascular and all-cause mortality. Testing for the proportional hazards assumption did not yield statistically significant model violations (*P* = 0.11).

In the model of combined effects, we created 12 paired groups of alcohol consumption and resting heart rate status featuring all of the different combinations. The combination of non-drinking and heart rate of 61–79 bpm was considered the reference group. The multiplicative interaction of alcohol consumption and resting heart rate status on cardiovascular and all-cause mortality was evaluated with the likelihood ratio test, comparing the Cox regression model with only the main effects and the model that included both the main effects and the interaction terms. Hazard ratios (HRs) and 95% confidence intervals (CIs) of cardiovascular and all-cause mortality were derived. All analyses were performed using SAS Windows Version 9.3 (Cary, NC, USA), with two-tailed tests at the significance level of 0.05.

## RESULTS

### Sample characteristics

General characteristics of the study participants according to resting heart rate categories are presented in Table 1. The mean age of the participants was 66.2 (± 7.2) years, and the average resting heart rate was 73.3 (± 9.7) bpm. Participants with resting heart rates greater than 80 bpm had significantly higher systolic blood pressure (*P* < 0.001) and were also more likely to be cigarette smokers and heavy binge drinkers compared with the resting heart rate 61–79 bpm (reference group) category. Among the participants, 91.8% had received no formal education or had been educated only at an elementary school level. In the 20.8-year follow-up period, 1990 men (76.5%) died. These people represented 78.4% of those with resting heart rate lower than 60 bpm, 75.5% of those with resting heart rates 61–79 bpm, and 78.9% of those with resting heart rates greater than 80 bpm.

**Table 1.** Baseline characteristics by heart rate category of the male study population recruited for the Kangwha Cohort Study in South Korea, 1985–2005

Variables	Heart rate category, bpm			

	≤60 (*n* = 222)	61–79 (*n* = 1754)	≥80 (*n* = 624)	*P*
Age (SD), year	66.6 (7.5)	66.2 (7.3)	66.3 (7.1)	0.74
SBP (SD), mm Hg	147.2 (32.1)	146.7 (30.5)	152.9 (31.1)	<0.001
DBP (SD), mm Hg	69.8 (22.7)	71.1 (19.0)	72.2 (21.4)	0.25
BMI (SD), kg/m^2^	23.1 (23.4)	22.4 (17.3)	23.0 (25.4)	0.77
Education, %				
None	41.4	39.7	41.5	0.70
Elementary	52.3	51.7	50.8	
Higher	6.3	8.6	7.7	
Smoking, %				
Never	20.3	19.3	15.2	<0.001
Former	10.4	7.3	5.8	
Current				
1–19 cigarettes/day	28.4	25.7	26.6	
≥20 cigarettes/day	41.0	47.8	52.4	
Alcohol drinking, %				
Non-drinking	40.1	36.6	26.4	<0.001
Non-binge	46.9	44.0	47.6	
Moderate binge (6–11 drinks)	11.7	15.9	21.5	
Heavy binge (≥12 drinks)	1.4	3.5	4.5	
Occupation, %				
Agriculture	83.3	85.9	85.7	0.76
Other	16.7	14.1	14.3	
Chronic disease, %				
Ever	49.1	41.9	48.2	<0.001
Never	50.9	58.1	51.8	
Death, %				
Yes	78.4	75.5	78.9	<0.001

BMI, body mass index; bpm, beats per minute; DBP, diastolic blood pressure; SBP, systolic blood pressure; SD, standard deviation.

### Alcohol consumption and mortality

The association of alcohol consumption with the risk of dying from cardiovascular and all-cause mortality is presented in Table 2. Men who reported heavy binge drinking had consistently higher risk of cardiovascular and all-cause mortality than men who reported non-drinking or non-binge drinking. Adjusting only for age (model 1), alcohol consumption was positively associated with the risk of death, showing a gradual increase in cardiovascular and all-cause mortality with increasing alcohol consumption (Table 2). In the highest quartile (heavy binge drinking), the age-adjusted HRs were 1.96 (95% CI, 1.16–3.32) and 1.53 (95% CI, 1.21–1.95) for cardiovascular and all-cause mortality, respectively, compared to the reference group (non-drinkers). After adjustment for age and other risk factors (model 2), the HRs were reduced only slightly and remained significant: 1.91 (95% CI, 1.12–3.25) for cardiovascular and 1.42 (95% CI, 1.12–1.82) for all-cause mortality (Table 2). The association between binge drinking and deaths from CVD were stronger than all-cause mortality in both models (Table 2).

**Table 2.** Hazard ratios (HR) for cardiovascular and all-cause mortality according to alcohol drinking and heart rate in the Kangwha Cohort Study in South Korea, 1985–2005

Alcohol drinking and resting heart rate	Cardiovascular mortality				All-cause mortality			
	
	Model 1^a^		Model 2^b^		Model 1^a^		Model 2^b^	
			
	HR	(95% CI)	HR	(95% CI)	HR	(95% CI)	HR	(95% CI)
Alcohol drinking								
Non-drinking	1.00	(Reference)	1.00	(Reference)	1.00	(Reference)	1.00	(Reference)
Non-binge	1.16	(0.90–1.48)	1.13	(0.88–1.46)	1.14	(1.08–1.26)	1.09	(0.99–1.21)
Moderate binge (6–11 drinks)	1.23	(0.90–1.69)	1.22	(0.89–1.68)	1.11	(0.97–1.26)	1.04	(0.91–1.19)
Heavy binge (≥12 drinks)	1.96	(1.16–3.32)	1.91	(1.12–3.25)	1.53	(1.21–1.95)	1.42	(1.12–1.82)
Heart rate (bpm)								
Heart rate ≤60 bpm	1.06	(0.88–1.28)	1.07	(0.89–1.29)	1.08	(0.92–1.26)	1.08	(0.92–1.26)
Heart rate 61–79 bpm	1.00	(Reference)	1.00	(Reference)	1.00	(Reference)	1.00	(Reference)
Heart rate ≥80 bpm	1.35	(1.20–1.51)	1.33	(1.18–1.49)	1.16	(1.05–1.29)	1.13	(1.02–1.26)

^a^Adjusted for age.

^b^Adjusted for age, hypertension, body mass index, education, smoking, occupation, chronic disease and antihypertensive medication.

bpm, beats per minute; CI, confidence interval.

### Heart rate categories and mortality

The 61–79 bpm category served as the reference group for resting heart rate. In the age-adjusted analysis, resting heart rate greater than 80 was significantly associated with cardiovascular and all-cause mortality. The HR for dying from CVD was 1.35 (95% CI, 1.20–1.51), while that for all-cause mortality was 1.16 (95% CI, 1.05–1.29). Adjusting for all covariates, the HRs were reduced only slightly (cardiovascular mortality, HR 1.33 [95% CI, 1.18–1.49]; all-cause mortality, HR 1.13 [95% CI, 1.02–1.26]) compared to the age-adjusted analysis, but remained significant. The magnitude of the HRs was higher for cardiovascular mortality than all-cause mortality.

### Combined effects of alcohol consumption and heart rate

Table 3 shows the risk of cardiovascular and all-cause mortality according to the combination of alcohol consumption and resting heart rate categories. The group composed of non-drinking subjects with resting heart rate 61–79 bpm was the reference group. The highest risk for cardiovascular and all-cause mortality was observed in the group with heavy binge drinking (≥12 drinks on one occasion) and highest resting heart rate (≥80 bpm). This group had a 2.35-fold increase in risk of death from CVD compared to the reference group. After adjusting for age, the HRs of cardiovascular mortality for the group with resting heart rate greater than 80 bpm were 1.47 (95% CI, 1.18–1.84) for non-drinkers, 1.60 (95% CI, 1.34–1.92) for non-binge drinkers, 1.79 (95% CI, 1.43–2.25) for moderate binge drinkers, and 2.35 (95% CI, 1.54–3.59) for heavy binge drinkers. Adjusting for all covariates, the HRs were reduced only slightly but remained significant. Elevated heart rate also strengthened the association between heavy binge drinking and cardiovascular mortality, with the highest mortality risk observed among the heavy binge drinkers with a resting heart rate greater than 80 bpm (HR 2.25, 95% CI, 1.47–3.45). On testing for interaction, however, results were not significant (*P* = 0.67). The HRs of cardiovascular and all-cause mortality for the heavy binge drinkers with resting heart rate lower than 60 bpm were 5.07 (95% CI, 1.25–20.45) and 4.25 (95% CI, 1.36–13.26), respectively (Table 3).

**Table 3.** Combined effects of heart rate and alcohol drinking on cardiovascular and all-cause mortality in the Kangwha Cohort Study in South Korea, 1985–2005

Alcohol drinking and resting heart rate	Cardiovascular mortality					All-cause mortality				
	
	*n*	Model 1^a^		Model 2^b^		*n*	Model 1^a^		Model 2^b^	
			
		HR	(95% CI)	HR	(95% CI)		HR	(95% CI)	HR	(95% CI)
Heart rate ≤60 bpm										
Non-drinking	11	1.16	(0.86–1.56)	1.15	(0.85–1.55)	65	1.06	(0.81–1.37)	1.06	(0.81–1.37)
Non-binge	10	1.43	(1.08–1.88)	1.32	(1.00–1.74)	89	1.34	(1.07–1.68)	1.26	(1.01–1.59)
Moderate binge (6–11 drinks)	4	1.15	(0.69–1.93)	1.09	(0.65–1.84)	17	0.96	(0.59–1.55)	0.87	(0.54–1.42)
Heavy binge (≥12 drinks)	2	5.30	(1.32–21.34)	5.07	(1.25–20.45)	3	4.49	(1.44–14.00)	4.25	(1.36–13.26)
Heart rate 61–79 bpm										
Non-drinking	80	1.00	(Reference)	1.00	(Reference)	485	1.00	(Reference)	1.00	(Reference)
Non-binge	102	1.35	(1.17–1.55)	1.30	(1.12–1.50)	581	1.15	(1.02–1.30)	1.10	(0.97–1.24)
Moderate binge (6–11 drinks)	37	1.32	(1.09–1.59)	1.26	(1.04–1.52)	206	1.14	(0.97–1.35)	1.07	(0.91–1.26)
Heavy binge (≥12 drinks)	9	1.72	(1.21–2.43)	1.51	(1.06–2.15)	52	1.61	(1.21–2.15)	1.48	(1.11–1.98)
Heart rate ≥80 bpm										
Non-drinking	18	1.47	(1.18–1.84)	1.36	(1.09–1.71)	136	1.28	(1.05–1.55)	1.22	(1.01–1.49)
Non-binge	37	1.60	(1.34–1.92)	1.52	(1.27–1.82)	236	1.28	(1.10–1.50)	1.21	(1.03–1.42)
Moderate binge (6–11 drinks)	20	1.79	(1.43–2.25)	1.71	(1.35–2.15)	100	1.26	(1.01–1.56)	1.16	(0.93–1.44)
Heavy binge (≥12 drinks)	7	2.35	(1.54–3.59)	2.25	(1.47–3.45)	20	1.47	(0.94–2.31)	1.37	(0.87–2.14)

^a^Adjusted for age.

^b^Adjusted for age, hypertension, body mass index, education, smoking, occupation, chronic disease and antihypertensive medication.

bpm, beats per minute; CI, confidence interval; HR, hazard ratio.

## DISCUSSION

We report findings of a survival analysis examining the combined effect of alcohol intake and resting heart rate on cardiovascular and all-cause mortality among Korean men. Our study showed that heavy binge drinking and elevated resting heart rate alone and in combination are strong predictors of cardiovascular and all-cause mortality. This association remained significant even after controlling for a number of factors including age, BMI, education, occupation, chronic disease, cigarette smoking, and hypertension. The association between alcohol consumption and resting heart rate demonstrated in the present study is in line with results from previous studies, which showed that alcohol drinking was associated with heart rate,^7^^,^^12^ particularly high resting heart rate.^8^^,^^11^

The present study showed that the HRs for cardiovascular and all-cause mortality were higher for individuals with coexisting heavy binge drinking and high resting heart rate than for either of those factors alone. The risk of death from CVD increased from 1.36 among individuals with resting heart rates greater than 80 bpm in the non-drinking alcohol consumption category to 1.52, 1.71, and 2.25 among individuals with resting heart rates greater than 80 bpm within the alcohol consumption categories non-binge, moderate binge, and heavy binge, respectively. The highest risk of cardiovascular mortality, with a 2.25-fold risk of death, was seen among those who were both heavy binge drinkers and had a resting heart rate greater than 80 bpm, although no multiplicative interaction was observed. There was some indication that heavy binge drinkers with a resting heart rate 60 bpm or below had an increased mortality risk. Bradycardia, especially in older people, may facilitate heart failure through inadequate cardiac output or by inducing increased ventricular diastolic pressures.^15^ Our findings support other prospective studies with community-based samples showing that binge drinking and a high heart rate are strong predictors of cardiovascular death in men.^4^^,^^10^

Binge drinking has been reported to increase CVD risk, such as sudden death, myocardial infarction, mortality after myocardial infarction, and stroke.^16^ One of the pathways by which binge drinking leads to CVD may be by increasing the risk and progression of atherosclerosis. The odds of coronary calcification, a marker of atherosclerosis, were doubled among binge drinkers compared to non-binge drinkers.^17^ In addition, binge drinking is associated with macrovascular and microvascular endothelial dysfunction, an early indicator of atherosclerosis.^16^ Measurements of plaque height and intima-media thickness of the carotid artery of middle-aged men revealed accelerated atherosclerosis for those drinking ≥6 drinks per occasion.^18^ Binge drinking may also contribute to sudden death caused by ventricular arrhythmias. Acute and excessive ingestion of alcohol has been demonstrated to induce QTc prolongation associated with ventricular tachyarrhythmias and sudden cardiac death in healthy adults.^19^ Binge drinking appears to lower the threshold of ventricular fibrillation, predisposing myocardial infarction survivors to an increased risk of sudden cardiac death.^20^ Binge drinking has also been demonstrated to enhance thrombosis and inhibit fibrinolysis by increasing platelet reactivity, thromboxane B_2_ formation, and plasminogen activator inhibitor-1 activity. Further, rapid changes in blood pressure, with increases in both systolic and diastolic blood pressure after binge drinking, followed by a decrease below normal levels when alcohol concentration falls, have been observed among binge drinkers, increasing the likelihood of strokes.^21^

Elevated resting heart rate has been shown to increase the risk of incident cardiovascular mortality.^9^ The exact mechanism through which resting heart rate influences adverse cardiovascular events is still unclear, but possible explanations include predisposition to ischemia and arrhythmia, increased risk of plaque rupture due to hemodynamic forces, and acceleration of atherosclerosis by increasing sheer stress to arterial walls. Resting heart rate may also be a marker for increased sympathetic activity, contributing to an increased CVD risk.^22^ Elevated heart rate would lead to increased myocardial oxygen demand and energy depletion. Increased sympathetic tone has also been shown to be linked to insulin resistance and metabolic syndrome, with increased triglycerides, body mass index, and total cholesterol.^23^ More recently, systemic inflammation and endothelial dysfunction have been implicated in the association between increased resting heart rate and incident heart failure and cardiovascular mortality.^24^

There are several limitations of this study. First, alcohol consumption was based on self-reports of people aged 55 or older and may not accurately reflect the actual intake. However, both the short-term and long-term recalls of alcohol intake have been reported to be valid and reliable.^25^ A second interview conducted with 3381 survivors in 1994 showed percent agreement between drinking status in 1985 and 1994 to be 87% and Cohen’s kappa value 0.70, demonstrating substantial agreement. Second, despite the relatively large sample size, some of the subgroups included a comparatively small number of individuals. For example, the highest HR for males who were both heavy binge drinkers and had a resting heart rate lower than 60 bpm was found in a group of only two or three individuals. The substantial difference in heart rate between this group and the others could be due to the small group size. Third, we had no information about some important risk factors for CVD, such as being former drinkers. Thus, we cannot exclude the possibility that these factors might have modified the effects of elevated heart rate and binge drinking on cardiovascular and all-cause mortality, and the possibility of residual confounding related to these issues remains. Fourth, the physical activity of participants was not evaluated. Because many of the participants (82.8%) were engaged in agriculture, we used occupation as a proxy measure for physical activity. Finally, the study population came from a rural region in Korea and the results may not be generalizable to an urban setting or other Asian populations.

In the present study, compared with that of nondrinkers, mortality risks from CVD and all-cause were highest in older heavy binge drinkers having ≥12 drinks on one occasion. Such a relationship markedly increased when heavy binge drinkers had elevated resting heart rate greater than 80 bpm. These findings also suggest that an elevated resting heart rate should not be regarded as a less serious risk factor in heavy binge drinking than in non-drinking older men. It may be important to closely monitor heart rates among those with a tendency to binge drink to help prevent cardiovascular and all-cause deaths.
